# Owners’ Perceptions of Pet influence: Relation to Health Outcomes & Pet Attachment in Community-Living Older Adults

**DOI:** 10.1093/geroni/igaa057.3398

**Published:** 2020-12-16

**Authors:** Erika Friedmann, Nancy Gee, Eleanor Simonsick, Barbara Resnick, Erik Barr, Melissa Kitner-Triolo, Alisha Hackney

**Affiliations:** 1 University of Maryland School of Nursing, Baltimore, Maryland, United States; 2 Virginia Commonwealth University, Richmond, Virginia, United States; 3 National Institute on Aging, Bethesda, Maryland, United States

## Abstract

Pet ownership (PO) has been linked to better health outcomes in older adults, particularly those with chronic health conditions. It is suggested that pets influence their owners lives both by encouraging social interaction and by interfering with owners’ willingness or ability to seek care for themselves. We use data from 6 questions about the positive and negative influence of pets on community dwelling older adults’ administered to pet owners (N=223, age >=50 years) in the Baltimore Longitudinal Study of Aging. We use principal components analysis (oblique rotation) to extract dimensions of owner’s perceptions of pet influences (PPI) and examine the relationship of these dimensions to owners’ cognitive, physical functional, and psychological status. Three dimensions of PPI include: fiscal/health challenges (F1: 3 items, alpha=0.70), wellness promotion (F2: 2 items, alpha=0.80); and reason for social/travel constraints (F3: 1 item). In regression analysis with all factors entered simultaneously, after controlling for age, higher magnitude of F1 significantly independently predicted poor physical quality of life (p=.0007), greater perceived stress (p=0.041), and lower happiness (p=0.014); F2 did not independently predict any health outcome; higher F3 significantly independently predicted lower emotional vitality (p=0.048). Controlling for age, all three factors were independent predictors of pet attachment (p’s=0.001, 0.010, 0.047, respectively). F1 and F3 were positively and F2 was negatively correlated with attachment. PPI was associated with owners’ physical and mental health. Perhaps older adults with higher attachment to pets are more likely to keep them despite higher challenges.

